# Mercury in the retina and optic nerve following prenatal exposure to mercury vapor

**DOI:** 10.1371/journal.pone.0220859

**Published:** 2019-08-07

**Authors:** Roger Pamphlett, Stephen Kum Jew, Svetlana Cherepanoff

**Affiliations:** 1 Discipline of Pathology, Sydney Medical School, Brain and Mind Centre, The University of Sydney, Sydney, Australia; 2 Department of Neuropathology, Royal Prince Alfred Hospital, Sydney, Australia; 3 Sydpath, St Vincent's Hospital, Sydney, Australia; 4 Northern Clinical School, The University of Sydney, Sydney, Australia; 5 St Vincent’s Clinical School, University of New South Wales, Sydney, Australia; National Center for Toxicological Research, UNITED STATES

## Abstract

Damage to the retina and optic nerve is found in some neurodegenerative disorders, but it is unclear whether the optic pathway and central nervous system (CNS) are affected by the same injurious agent, or whether optic pathway damage is due to retrograde degeneration following the CNS damage. Finding an environmental agent that could be responsible for the optic pathway damage would support the hypothesis that this environmental toxicant also triggers the CNS lesions. Toxic metals have been implicated in neurodegenerative disorders, and mercury has been found in the retina and optic nerve of experimentally-exposed animals. Therefore, to see if mercury exposure in the prenatal period could be one link between optic pathway damage and human CNS disorders of later life, we examined the retina and optic nerve of neonatal mice that had been exposed prenatally to mercury vapor, using a technique, autometallography, that detects the presence of mercury within cells. Pregnant mice were exposed to a non-toxic dose of mercury vapor for four hours a day for five days in late gestation, when the mouse placenta most closely resembles the human placenta. The neonatal offspring were sacrificed one day after birth and gapless serial sections of formalin-fixed paraffin-embedded blocks containing the eyes were stained with silver nitrate autometallography to detect inorganic mercury. Mercury was seen in the nuclear membranes of retinal ganglion cells and endothelial cells. A smaller amount of mercury was present in the retinal inner plexiform and inner nuclear layers. Mercury was conspicuous in the peripapillary retinal pigment epithelium. In the optic nerve, mercury was seen in the nuclear membranes and processes of glia and in endothelial cells. Optic pathway and CNS endothelial cells contained mercury. In conclusion, mercury is taken up preferentially by fetal retinal ganglion cells, optic nerve glial cells, the retinal pigment epithelium, and endothelial cells. Mercury induces free radical formation, autoimmunity, and genetic and epigenetic changes, so these findings raise the possibility that mercury plays a part in the pathogenesis of degenerative CNS disorders that also affect the retina and optic nerve.

## Introduction

The optic pathway is a frequent site of pathology in human central nervous system (CNS) diseases, with retinal atrophy being described in people with Alzheimer disease, Parkinson disease and amyotrophic lateral sclerosis [1]. Optic neuritis is a common early manifestation in multiple sclerosis, and retinal atrophy occurs in multiple sclerosis without the presence of optic neuritis [2]. In all these disorders, thinning of the inner retina appears to be the most prominent feature, usually because of atrophy of the nerve fibre layer [1]. The retina and optic nerve are extensions of the central nervous system, so optic pathway changes may be secondary to CNS damage. On the other hand, the initiating pathogenetic agent that triggers the disorder in the brain could also involve the optic pathway, since eye signs are present before damage to the brain becomes apparent [1]. There is increasing interest in the concept the early life exposures to toxicants influence the onset of later life disorders [3], probably through epigenetic mechanisms [4]. A study of the retina and optic nerve in fetal mice that have been exposed to toxic environmental agents could therefore give clues to the underlying pathogenesis of these disorders.

Toxic metals such as mercury have been implicated in neurological disorders, and could act in combination with genetic or other susceptibilities to cause widespread cell damage [5,6]. In addition, mercury has been thought to be responsible for several human retinal disorders [7,8,9]. Mercury has been located in the adult primate retina and optic nerve after exposure to mercury vapor [10]. This led us to wonder if mercury localises to the same regions in the fetus, where it might lead to genetic or epigenetic changes that predispose to later-life disorders [11]. Mercury vapor crosses the rodent placenta readily, and rodents in late gestation develop a chorioallantoic placenta like that of humans [12]. Furthermore, human exposure to mercury during pregnancy is common, mostly from fish consumption or from mercury-containing amalgam dental restorations, and mercury in human maternal blood crosses the placenta to enter the fetal circulation [13]. We therefore exposed mice in late pregnancy to non-toxic levels of mercury vapor and used a histochemical method, autometallography, to look for the presence of mercury in the eyes, optic nerves and brains of their neonatal offspring.

## Materials and methods

### Study design

Tissue blocks from a project that studied the effect of transplacental mercury on the developing brain and spinal cord [12] were sectioned to examine the eyes and optic nerves. Briefly, over gestational days 14–18, four C57 pregnant mice were exposed to a non-toxic dose of 0.5 mg/m^3^ of mercury vapor for 4 hours a day. One pup from each litter was sacrificed with carbon dioxide on postnatal day 1 and fixed in 10% formalin. Serial 3 mm blocks of tissue were taken throughout the head and body and processed routinely for embedding in paraffin wax. Control pups with no fetal mercury exposure were sacrificed at the same time and treated in the same manner. In paraffin blocks containing the eyes, a 7 μm section was first cut and stained with hematoxylin, followed by 7 μm gapless serial sections throughout the whole block, all stained with silver nitrate autometallography (AMG) to detect inorganic mercury attached to sulphides or selenides, which is visible microscopically as black silver-coated mercury grains [14]. In each staining run, a mouse spinal cord section where motor neuron cell bodies were known to contain mercury after an intraperitoneal injection of mercuric chloride [15] was included as a positive control.

### Ethics

The methods to expose mice to mercury vapor, animal housing, handling, sacrifice, and tissue preparation had been approved by the University of Sydney Animal Ethics Committee [12]. Because this work was undertaken on archived paraffin tissue blocks the ethics committee waived the requirement for renewal of the ethics protocol.

## Results

### Clinical

Dams, and other offspring exposed to the same dose of mercury vapor but not sacrificed, suffered no ill-effects, and fed, moved, behaved, and gained weight normally [12].

### The neonatal mouse retina

**Fig 1:**
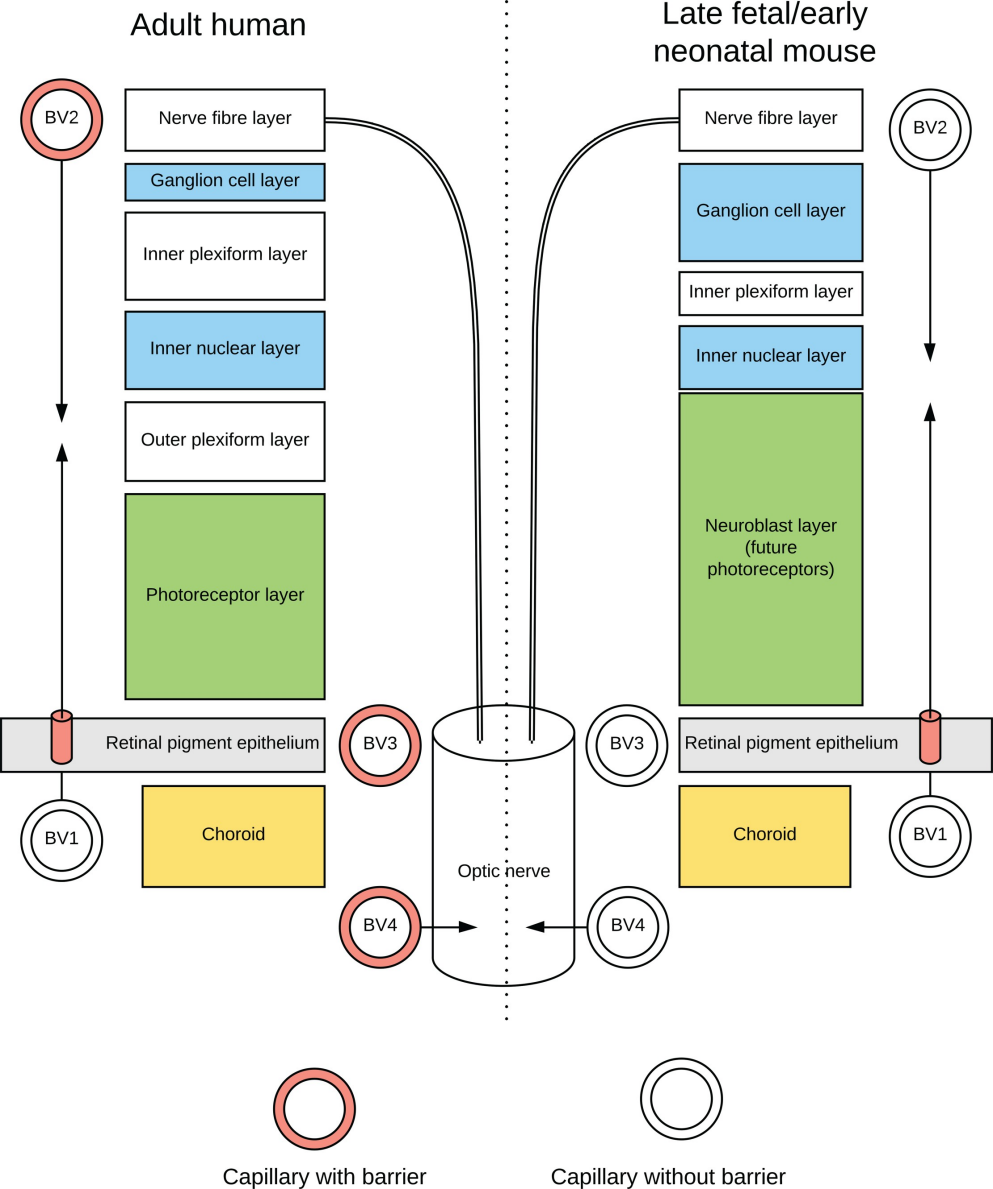
Diagram comparing eye anatomy of the adult human and neonatal mouse. The outer retinal layers are supplied by blood vessels (BV1) from the posterior ciliary arteries, terminating in the choriocapillaris, a fenestrated capillary network without tight junctions. An outer blood-retinal barrier is formed by tight junctions in the retinal pigment epithelial cells, after which oxygen is supplied by diffusion to the outer retina. An inner blood-retinal barrier is formed by adult capillary endothelial cells with tight junctions (BV2, filled walls) originating from the central retinal artery. The optic nerve head is supplied primarily by a vascular circle (BV3) from the posterior ciliary arteries, and from the peripapillary choroid vessels. The body of the optic nerve is supplied from the ophthalmic artery via the pial plexus of blood vessels which penetrate the nerve (BV4). The endothelial cells of adult optic nerve capillaries have tight junctions, forming a blood-optic nerve barrier. Capillaries of the inner retina, and possibly the optic nerve, have limited blood-retina and blood-optic nerve barriers (unfilled walls) in the late fetal mouse.

The neonatal mouse eye is relatively underdeveloped (the eye only opens on postnatal day 9) so there are several anatomical differences between it and a human adult eye [16]. However, the origins of the adult retinal layers are seen in the neonate (Fig 1). The neonatal mouse has an incomplete inner blood-retinal barrier since transcytosis is only suppressed from postnatal day 10 onwards [17].

**Fig 2:**
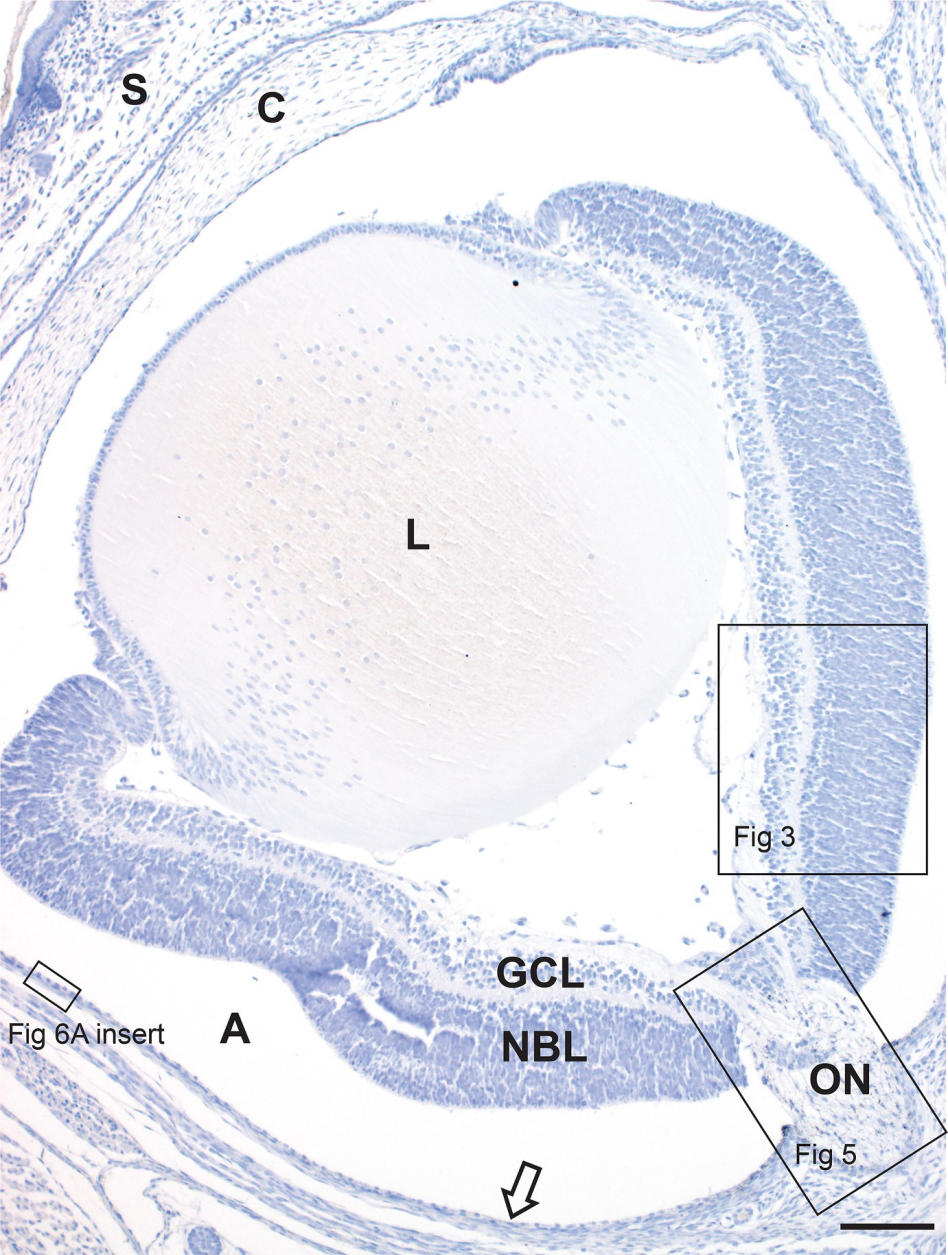
The neonatal mouse eye. Skin (S) covers the eye of the neonatal mouse. The ganglion cell layer (GCL) and neuroblast layer (NBL) of the retina are separated by a pale inner plexiform layer (for more detail see Fig 3). The optic nerve (ON) is seen at the lower right of the image (for more detail see Fig 5). The retinal pigment epithelium (arrow) is separated from the rest of the retina by an artefactual space (A). C: Cornea, L: lens. AMG/hematoxylin, Bar = 100 **μ**m.

**Fig 3:**
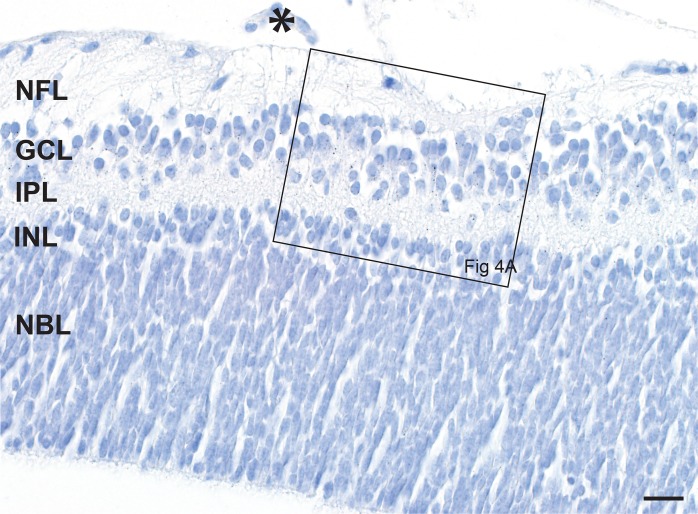
The neonatal mouse retina. Ganglion cells in the ganglion cell layer (GCL) are numerous in the neonatal mouse, and the nerve fibre layer (NFL) is prominent. Involuting hyaloid vessels (asterisk) are seen on the surface of the retina. The future inner nuclear layer (INL) is three-to-four cells thick below the inner plexiform layer (IPL), with slightly paler nuclei (best seen in Fig 4B) than the underlying neuroblast layer (NBL), the source of the photoreceptors. At this magnification black AMG grains are just visible in the ganglion cell layer (see Fig 4A for a high power view). AMG/hematoxylin, Bar = 20 **μ**m.

**Fig 4:**
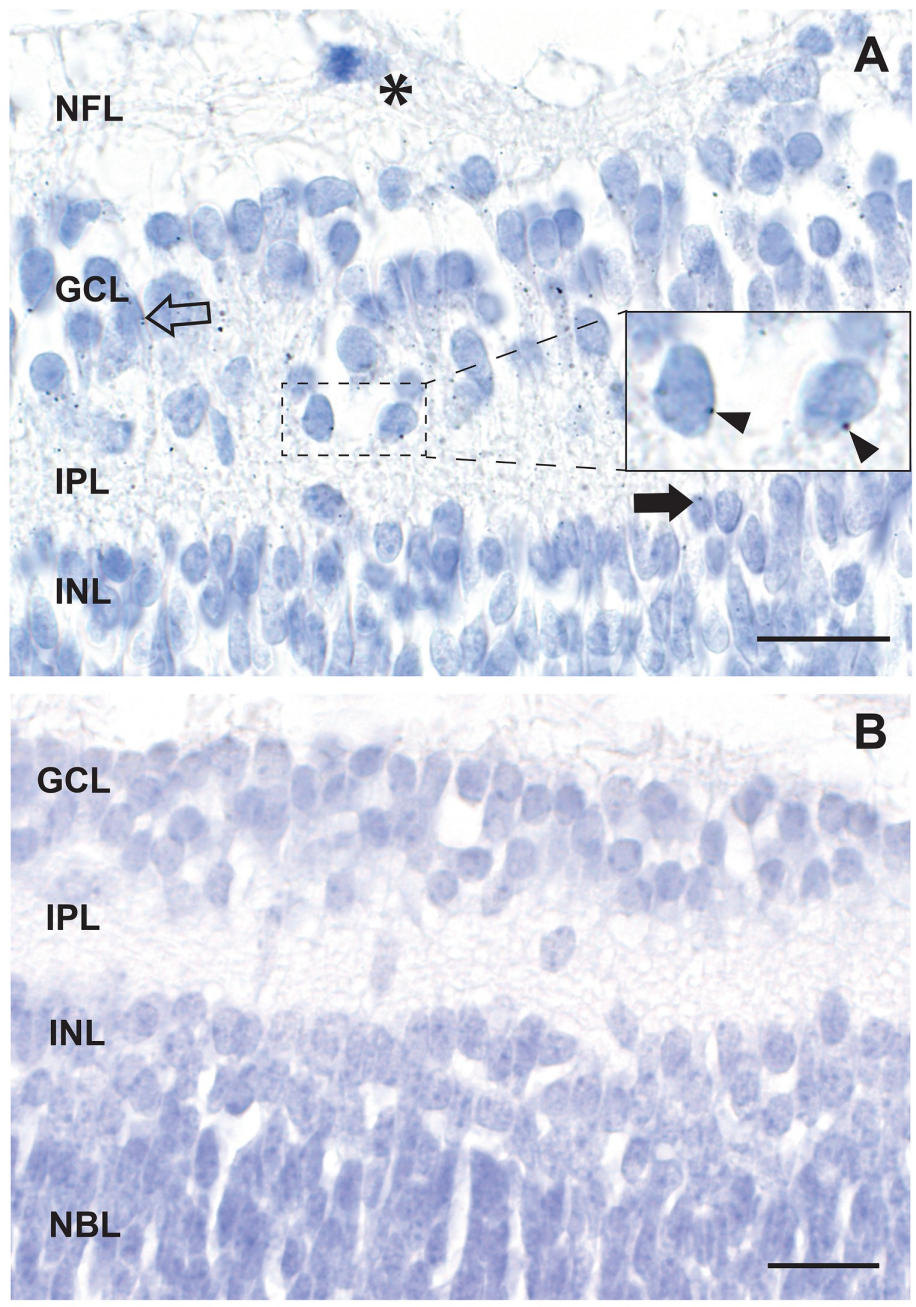
Mercury in the inner retina. A. AMG grains are attached to the nuclear membranes of scattered ganglion cells (eg, arrowheads in inset) and in endothelial cells (open arrow). A few AMG grains are present in the inner plexiform layer, and in nuclear membranes (eg, closed arrow) and endothelial cells of the inner nuclear layer. A mitosis (asterisk) is present in the nerve fibre layer. B. No AMG grains are seen in the cells of the inner retina in a neonatal mouse that had not been exposed to prenatal mercury vapor. NFL: nerve fibre layer, GCL: ganglion cell layer, IPL: inner plexiform layer, INL: inner nuclear layer, NBL: neuroblast layer. AMG/hematoxylin, Bars = 20 μm.

The anatomy of the neonatal mouse eye and optic nerve head is seen in Fig 2. At postnatal day 1 the immature mouse eye is still covered by skin. The ganglion cells are numerous but undeveloped. The inner neuroblast layer (the future inner nuclear layer) is a layer of slightly paler-staining cells adjacent to the outer neuroblastic layer, where the photoreceptors form (Figs 3 and 4B) [16].

### Mercury in the neonatal mouse retina

Mercury staining was visible in the ganglion cell layer, where it was attached to the nuclear membranes of the ganglion cells and in endothelial cells (Fig 4A). Scattered mercury staining was present in the nerve fibre and inner plexiform layers, and in nuclear membranes and capillary walls of the inner nuclear layer. Mercury in the inner retina cells was most prominent at the posterior pole of the eye, with decreasing staining with increasing distance from the posterior pole, until no mercury was seen in cells at the anterior pole. No mercury was seen in the outer neuroblast (future photoreceptor) layer. No other areas of the eye, including the anterior segment, contained mercury. No black grains were seen in any eyes or optic nerves of mercury-exposed mice that were stained with hematoxylin only. No AMG grains were seen in the retina of mice that had not been exposed to prenatal mercury vapor (Fig 4B).

**Fig 5:**
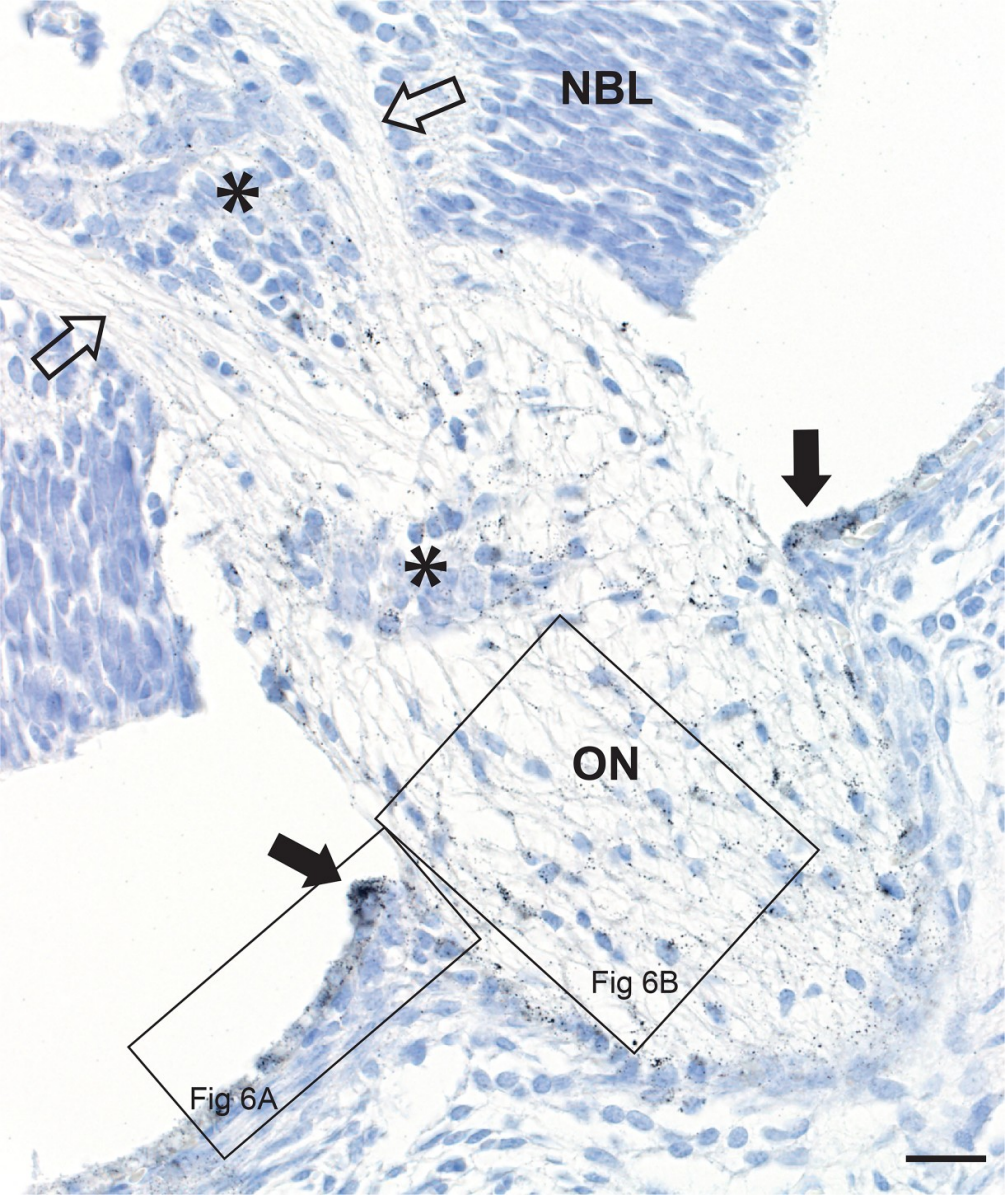
Mercury in the retinal pigment epithelium and optic nerve head. In a mouse exposed to mercury vapor, two nerve fibre bundles (open arrows) descend from the retinal nerve fibre layer into the optic nerve (ON) head. Black AMG grains can be seen in the retinal pigment epithelium (closed arrows) adjacent to the optic nerve head (see Fig 6A for high power view), and within the optic nerve head itself (see Fig 6B for high power view). Remnants of hyaloid blood vessels are present (asterisks). NBL: neuroblast layer. AMG/hematoxylin, Bar = 20 μm.

**Fig 6:**
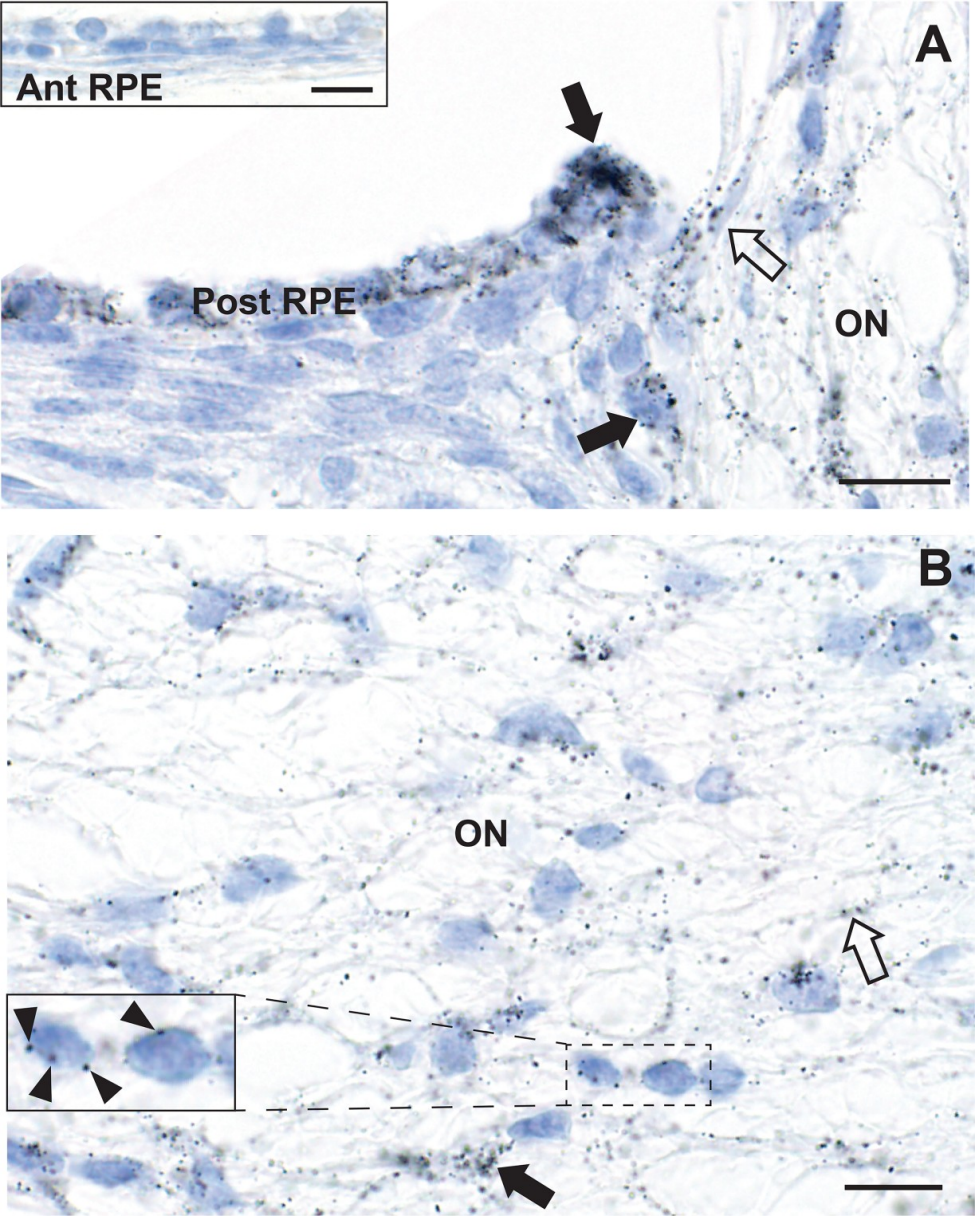
Mercury in the peripapillary retinal pigment epithelium and optic nerve head. A. Numerous AMG grains (closed arrows) are present in the peripapillary posterior retinal pigment epithelium (Post RPE), with decreasing amounts of mercury in the epithelium as the distance from the optic nerve (ON) head increases. AMG grains are seen in a capillary at the edge of the optic nerve (open arrow) and within the optic nerve head itself. Inset: no AMG grains are seen in the anterior retinal pigment epithelium (Ant RPE) of the same mouse (see Fig 2 for location). B. In the optic nerve head, AMG grains are attached to glial cell nuclear membranes (eg, arrowheads in the inset), glial cell processes (eg, closed arrow), and endothelial cells (eg, open arrow). AMG/hematoxylin, Bars = 10 μm.

In neonatal mice exposed to prenatal mercury vapor, dense mercury staining was present in the peripapillary retinal pigment epithelium (Fig 5), with decreasing amounts of mercury in pigment epithelial cells further from the optic nerve head (Fig 6A). No mercury was seen in the anterior retinal pigment epithelium (inset in Fig 6A).

### Mercury in the neonatal mouse optic nerve

**Fig 7:**
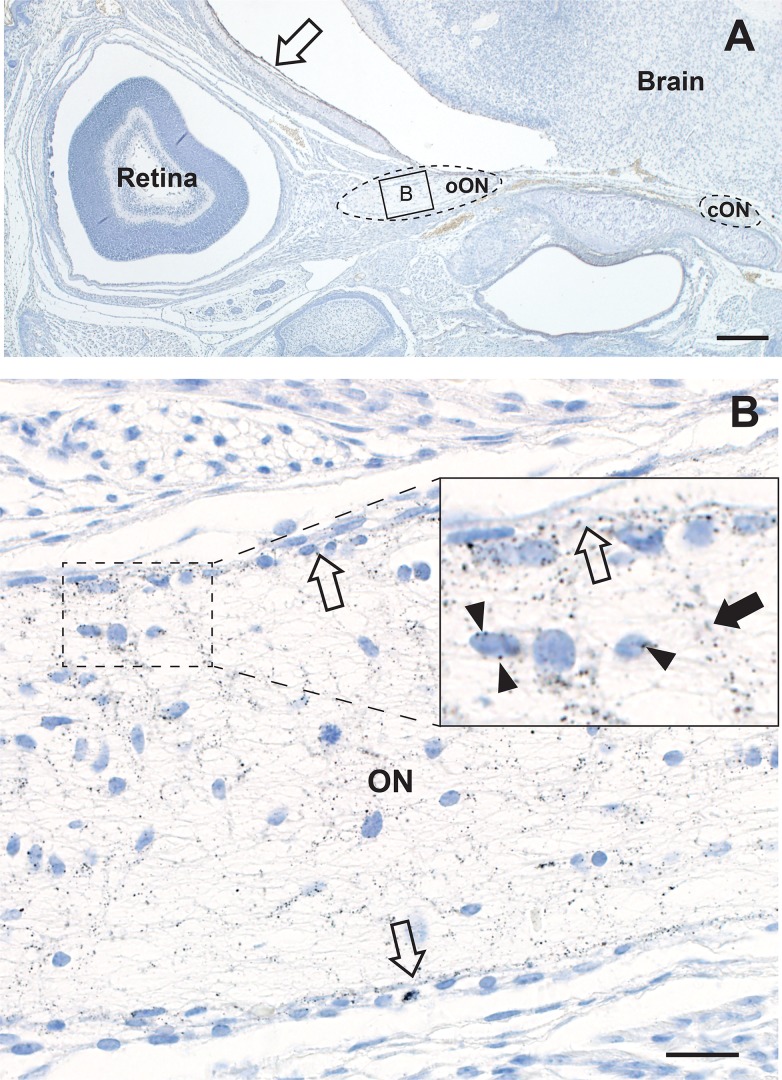
Mercury in the intra-orbital and intracranial optic nerves. A. The intra-orbital optic nerve (oON, dotted oval on left, box is at high power in panel B below) from one eye, and the intracranial optic nerve (cON, dotted oval on right) from the other eye, are present. The intracranial meningeal cells (open arrow) contain AMG grains. AMG/hematoxylin, Bar = 200 μm. B. The intraorbital optic nerve contains numerous AMG grains, both in glial nuclear membranes (eg, arrowheads in inset) and in processes. AMG grains are prominent in endothelial cells of the peripheral pial plexus (all open arrows) and their branches (closed arrow in inset) that penetrate the optic nerve. AMG/hematoxylin, Bar = 20 μm.

Mercury staining was prominent in the optic nerve head, where it was present in glial cell nuclear membranes and processes, as well as in endothelial cells (Fig 6B). Mercury was seen in same cells in the intra-orbital and intracranial optic nerves (Fig 7A), as well as in endothelial cells of the pial vascular plexuses supplying the bodies of the optic nerves (Fig 7B).

### Mercury in the neonatal brain endothelial cells and meninges

**Fig 8:**
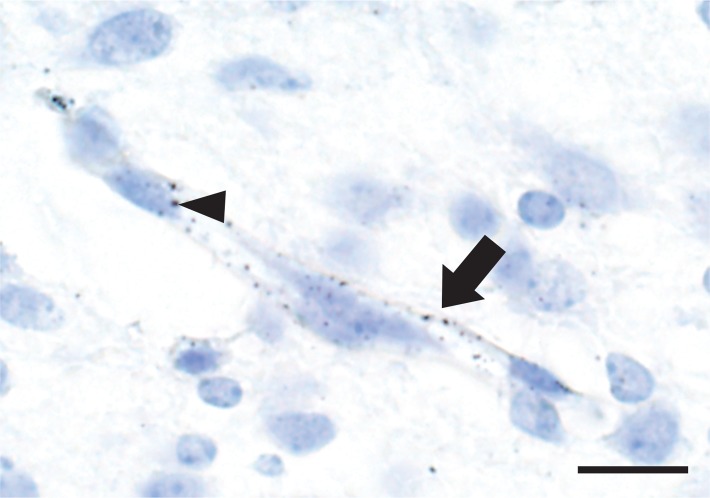
Mercury in brain endothelial cells. AMG grains are present in the endothelial cells (arrow) of a capillary within the thalamus. An occasional AMG grain is attached to an endothelial nuclear membrane (arrowhead). Surrounding neurons and glia do not contain mercury. AMG/hematoxylin, Bar = 10 μm.

Mercury was seen in the brain leptomeninges (Fig 7A) and in scattered endothelial cells of capillaries within the brain parenchyma, where the mercury appeared to be attached to the nuclear membranes (Fig 8). No mercury was seen in the processes, perikarya or nuclear membranes of brain neurons or glial cells, as noted previously [12].

### Neonatal mice not exposed to mercury

In neonatal mice that had not been exposed to prenatal mercury vapor, no AMG staining was seen in the inner retina (Fig 4B), retinal pigment epithelium, optic nerves or endothelial cells.

## Discussion

Key findings of this study were that after exposure to mercury vapor in late pregnancy, mercury was taken up preferentially in fetal retinal ganglion cells, the peripapillary retinal pigment epithelium, optic nerve glia and endothelial cells. Mercury appeared to have a predilection for the nuclear membrane of cells.

The distribution of mercury we found in the fetal retina and optic nerve is similar to that described in the adult squirrel monkey after exposure to mercury vapor, where autometallography was also used to demonstrate the presence of mercury within cells; mercury was still visible in these cells some years after exposure, indicating that these tissues retain mercury for long periods of time [10]. Mercury vapor also localises to the retina of the adult mouse eye [18], and to the retina of the adult rat and Marmoset monkey [19], while organic mercury localises to the optic nerve of larval zebrafish [20]. In these studies, however, the cellular distribution of the mercury could not be determined.

Unlike inorganic mercury, mercury vapor (with human exposure mostly from industrial occupations or mercury-containing dental amalgam restorations) and methylmercury (mostly from eating fish) both cross the placenta readily [21,22,23]. Methylmercury is slowly demethylated in fetal tissues into inorganic mercury [24]. Much of inhaled mercury vapor is oxidised in red blood cells to inorganic mercury by catalase, and inhibitors of catalase, such as ethanol, increase the amount of mercury vapor that is available to cross the placenta [25]. Mercury vapor from amalgam dental fillings may play a toxic role in the human retina, since people with amalgam fillings appear to have reduced volumes of the ganglion cell and inner plexiform layers (measured with optical coherence tomography) compared to controls [26]. Despite the different methods of absorption and excretion of the three forms of mercury [24], the tissue distribution of mercury in experimental animals is similar when administered as mercury vapor [19], organic mercury [27] or inorganic mercury [28].

Inorganic mercury is transported into the retinal pigment epithelium as a thiol-conjugate [29], which may help explain the marked uptake of mercury into the peripapillary retinal pigment epithelium in our mice. Since adult monkeys exposed to mercury vapor take up mercury into the retina and optic nerve [10], inner retinal cells and optic nerve glia may also contain the (as-yet unidentified) mercury transporters which are found in various cells throughout the body [30].

The mouse fetal retina may be more susceptible than the adult retina to mercury uptake since the blood-retinal barrier is still incomplete in the late fetal and early neonatal mouse [17] (Fig 1). Most of the developing mouse brain endothelial cells have developed blood-brain barriers by gestational day 15 [31], around the time our mice were exposed to mercury, but the blood-brain barrier develops at various times in the brain, so the optic nerve too may have an incomplete barrier at that time.

The location of mercury in our mouse eyes and optic nerves are in regions that are involved in Alzheimer disease, Parkinson disease and amyotrophic lateral sclerosis (Table 1). Environmental exposure to mercury has been suggested to be a risk factor for these disorders [5,6], and the pathogenetic mechanisms that appear to underlie these neurodegenerative diseases are those shared by mercury toxicity, ie, free radical formation [32], epigenetic alterations [11] and DNA damage causing somatic mutations [33]. Our finding that mercury attaches to the nuclear membrane of cells fits with the preference of mercury for membranous structures in electron microscopic studies of mice exposed to mercury, which is possibly due to the large numbers of mercury-binding sulfhydryl groups in these membranes [34]. Mercury damages proteins, so the location of mercury in the nuclear membranes may explain how decreased transport of substances in and out of the nucleus via nuclear pores could underlie some neurodegenerative disorders [35].

**Table 1:** Disorders with damage to the retina and optic nerve, compared to fetal uptake of mercury.

Disorder	Fetal mercury uptake
**Alzheimer disease/dementia**	
Thin retina [36,37,38,39,40,41,42]	Inner retina
Peripapillary retinal atrophy [36]	Peripapillary RPE
Optic nerve damage [43]	Optic nerve
**Parkinson disease**	
Inner retina damage [44]	Inner retina
**Amyotrophic lateral sclerosis**	
Thin retina [45,46], inner nuclear layer p62 inclusions [47]	Inner retina
**Multiple sclerosis**	
Inner nuclear layer damage [48]	Inner retina
Optic neuritis [49]	Optic nerve
**Age-related macular degeneration**	
Peripapillary retinal atrophy [50,51]	Peripapillary RPE
**Age-related peripapillary retinal atrophy**	
Peripapillary retinal atrophy [52]	Peripapillary RPE
**Neurodevelopmental disorders**	
Thin nerve fibre layer [53]	Inner retina

RPE: retinal pigment epithelium

The optic nerve is a frequent and early target for demyelination in multiple sclerosis [49], so here the preferential location of mercury in the optic nerve of our mice may be of relevance, since mercury provokes autoimmune reactions [54]. In addition, damage to the retinal inner nuclear layer has been described in multiple sclerosis [48], the same region in which mercury was found in our mice.

Age-related macular degeneration, a common cause of blindness in advancing age, has links with neurodegeneration since amyloid deposits like those in Alzheimer disease plaques are found in drusen, the sub-retinal epithelial cells seen in aging and macular degeneration [55]. Mercury is found in retinal pigment epithelial cells [19,29,56,57] and so may play a part in the pathogenesis of macular degeneration. In macular degeneration, free radical formation [58] and epigenetic variations [59], both toxic actions of mercury, have been postulated to underlie retinal damage. The mouse eye does not have a well-developed macula [60,61] and so is not an ideal model to study macular degeneration, but peripapillary atrophy and disruption of the retinal pigment epithelium are two features of macular degeneration [50,51] that could be explained by the uptake of mercury by the retinal pigment epithelium.

Peripapillary retinal atrophy alone is a frequent finding in aging [52]. The predilection in our mice for mercury to be taken up by the retinal pigment epithelium close to the optic nerve suggests that this region is particularly susceptible to toxic metals, and so may underlie this condition.

Endothelial cell uptake of mercury was prominent in the retina, optic nerve and brain of our fetal mice. The predisposition of endothelial cells to take up mercury has been noted before [62,63], and may lead to endothelial dysfunction [62,64]. This is of potential importance since mercury could play a role in the widespread dysfunction of the blood-brain barrier found in many adult neurodegenerative disorders [65,66,67] and in multiple sclerosis [68], and an increased permeability of brain capillaries might permit further toxicant entry into the brain with widespread consequences.

The present study has several limitations. (1) Mice were exposed to only one period of prenatal mercury exposure, in late gestation. This was because only in late gestation does the mouse placenta resemble that of the human chorioallantoic placenta, since the yolk sac supplies nutrients to the early mouse embryo until mid-gestation. Some toxins pass through the yolk sac that would be blocked by the chorioallantoic placenta, and some pass through the chorioallantoic placenta only, so studies on the early mouse embryo are not good models for human toxicity [69]. (2) We did not expose neonatal mice to the same prenatal dose of mercury vapor. However, when neonatal mice up to 10 postnatal days old were exposed to 10% of this dose, the distribution of mercury in the brain and spinal cord was the same as with the prenatal higher dose [12], suggesting the same pathways of mercury uptake are present in the late gestational and early postnatal time periods. (3) We were not able to assess the pathological consequences of mercury deposition in these cells, since all the gapless serial sections from each paraffin block were stained with autometallography, leaving no spare slides or tissue for analyses of mechanisms such as the generation of oxygen free radicals, autoimmune effects, or genetic or epigenetics changes. Free radical damage to cells containing mercury could be present in our mice, since we have shown oxidative damage to nucleic acids in the neurons of mice exposed to lower doses of mercury vapor than were used in the present experiment [70]. However, it is unlikely that pathogenic changes in this model would be seen so soon after mercury exposure, and in the absence of genetic susceptibilities that are thought to underlie human neurodegenerative disorders. (4) Autometallography demonstrates only inorganic mercury, and will not, for example, detect methylmercury. However, inorganic mercury is the proximate toxic agent for all forms of mercury [24], and so is arguably the most important form to identify to assess toxicity.

In conclusion, after pregnant mice are exposed to mercury vapor, mercury is taken up preferentially by the fetal inner retina, peripapillary retinal pigment epithelium, optic nerve and endothelial cells. This raises the possibility of a link between mercury exposure early in life and later-life neurodegenerative, demyelinating and retinal disorders in which these cells are affected. Epidemiological studies have so far not provided a convincing causal relationship between neurodegenerative diseases and toxic metals such as mercury from, for example, amalgam fillings [71,72], so advanced bioelemental mapping techniques on human eye and nervous system tissue will probably be required to obtain more evidence to support this hypothesis [73].
